# Design and psychometric properties of a questionnaire to assess gender sensitivity of perinatal care services: a sequential exploratory study

**DOI:** 10.1186/s12889-020-08913-0

**Published:** 2020-07-06

**Authors:** Masoumeh Simbar, Fatemeh Rahmanian, Soheila Nazarpour, Ali Ramezankhani, Narges Eskandari, Farid Zayeri

**Affiliations:** 1 Midwifery and Reproductive Health Research Center, School of Nursing and Midwifery, Shahid Beheshti University of Medical Sciences, Tehran, Iran; 2 Department of Midwifery and Reproductive Health, School of Nursing and Midwifery, Shahid Beheshti University of Medical Sciences, Tehran, Iran; 3 Department of Midwifery, Chalous Branch, Islamic Azad University, 17 Shahrivar Street, opposite the mosque of Imam Hussain (AS), Chalous, 4661961367 Iran; 4 Department of Public Health, Faculty of Health, Shahid Beheshti University of Medical Sciences, Tehran, Iran; 5 Department of Midwifery, School of Nursing and Midwifery, Qom University of Medical Sciences, Qom, Iran; 6 Department of Biostatistics, Faculty of Paramedicine, Shahid Beheshti University of Medical Sciences, Tehran, Iran

**Keywords:** Gender, Psychometric properties, Perinatal care, Questionnaire

## Abstract

**Background:**

Providing gender sensitive reproductive health service is recently emphasized by health organizations. This study aims to develop and assess psychometric properties of a questionnaire to assess gender sensitivity of perinatal care services (GS-PNCS) to be used by managers of perinatal services.

**Methods:**

This study is a mixed sequential (Qualitative-Quantitative) exploratory study. In the qualitative phase, 34 participants were interviewed and the items were generated. To evaluate the validity; face, content and construct validity were assessed. The reliability was assessed by internal consistency and stability calculation.

**Results:**

The content validity and reliability were demonstrated by S-CVR = 0.92 and S-CVI = 0.98, Cronbach’s α = 0.880 and the ICC = 0.980 to 0.947. Exploratory factor analysis showed 8 factors which explained more than 52.53% of the variance.

**Conclusion:**

GS-PNCS is a valid and reliable questionnaire, with 49 items to assess gender sensitivity of perinatal care services and helps health care managers and planners to improve the quality of the services.

## Background

Gender is a social construct referring to the culturally and historically based differences in the roles, attitudes and behaviors of men and women [1]. Men and women are not only different regarding their biological and hormonal conditions, vulnerability, prevalence and the incidence of diseases, but also respecting their health behaviors and experiences about diseases [2]. Besides, the social and economic status affect the responses of men and women to the diseases [3].

Gender sensitivity of health care services is fundamental for quality of care services and mentioned as a Global Strategy for Women’s Health [4]. It means that medical personnel understand gender health needs differences and use them in their decision making and activities in preventive and curative process [5]. The health care providers should consider the effects of biological factors of the individuals as well as clients’ position of life, position in the community and the social beliefs about femininity and masculinity [6–8].

Providing gender appropriate reproductive services are necessary because gender differences are effective on individuals’ reproductive health, especially on maternal health, contraceptive use, the prevention of high-risk sexual relationships, and the transmission of STIs [6]. Gender is one of the most important factors that must be taken into account when deciding about management and providing reproductive health care services [9].

Prenatal Care services (PNCS) are important programs in reproductive health, which aim to provide quality care and counseling for mothers to achieve women’s empowerment and rights [10]. However, the focus on women and the tendency to think that pregnancy, childbirth, child health and family planning are woman’s job caused men to be excluded from these services. While access to health services is one of the fundamental rights of both, men and women [11].

Development of indicators and tools to assess gender sensitivity of reproductive health including PNCS are essential for monitoring and evaluating of the services and improving the quality of care. Gender sensitive health policies and programs require a thorough analysis of needs to achieve women and men health [4, 12]. Reproductive health programs are not succeed if they would not be able to identify needs for gender sensitivity in their policies and implementation, and if they would not be able to response to the needs of women and men [13]. Therefore, a valid and reliable tool is necessary for health policy makers and managers to recognize gender sensitivity of reproductive health care and overcome the barriers and meet the needs to achieve quality reproductive health care services.

In this regard, a few tools were designed to measure gender sensitivity in STI services [14], male participation in PNCS [15] and reproductive health services [16]. The most comprehensive questionnaire to assess gender sensitivity in reproductive health services is available at the level of staff and facilities [17].

Since the causes of insensitivity of reproductive health services are complex and related to many other factors rather than health service providers and managers; such as institutional structure, values, priorities and process of providing services, the characteristics of health services and the culture of each country [17], this study aims to design a comprehensive, valid and reliable questionnaire to assess needs for providing the gender sensitive PNCS respecting all factors related to gender sensitivity of PNCS.

## Methods

This study was a mixed sequential exploratory study to develop a valid and reliable questionnaire to assess gender sensitivity of perinatal care services (GS-PNCS). So, the study was performed in two qualitative and quantitative phases, using Waltz steps [18] for tool development.

## The qualitative phase: development of the tool

### Design of the study

To generate appropriate preliminary items, an inductive-deductive approach was conduct. Firstly, a qualitative study with the content analysis approach was performed to explain the concept and dimensions of gender sensitive PNCS from the perspectives of the experts and providers. Then, a detailed related literature review was performed [19]. The items were extracted from both studies.

### The participants

The participants of the study were policy makers, providers and managers of PNCS including prenatal-, child birth and postpartum care services. The participants had at least 2 years of experience in PNCs and interest to participate in the study.

### Sampling

Sampling was started purposive and continued with a snowball sampling method. It was performed with the maximum diversity in gender, work experience and education. Finally, 34 policy makers, managers and service providers in PNCs participated in the study.

### Setting

All public and private clinics and hospitals in Shiraz and the headquarters of PNCS of Ministry of Health in Tehran were selected as the research environment.

### Tool of the study

The guide questions for the interview and data collection were: What is the concept of "gender sensitive PNCs?; What is your understanding and experience of the specific cultural, social and religious conditions that lead to the creation of different needs of women and men in PNCs?; What are the specific educational needs of the providers for a gender based counseling and care?

### Procedure of the study

Data was collected using a deep face to face individual interview by using the semi-structured interviews and continued until data saturation, when no new code of data was added to the study. The interviews were conducted by second author, Dr. Rahmanian who is PhD in Reproductive Health, and an assistant professor in the Department of Midwifery and Reproductive Health at Shiraz University of Medical Sciences. She has more than 15 years’ work experiences in perinatal care services as a midwife, trainer and manager. After introducing the interviewer, the participants were informed about goals of the study and confidentiality of their personal information. Also, field notes were made during and after interview. The interviews were performed after two pilot interviews. The average duration of interviews was 60 to 90 min. All interviews were audio recorded, transcribed, typed and coded on the same day. The transcripts were returned to participants for comments and corrections.

### Data analysis

The collected data was analyzed using qualitative conventional content analysis approach based on the Graneheim and Lundman’s method [20]. MAXQDA v.10 was used for data management.

### Data trustworthiness

Lincoln and Guba’s [21] criteria was considered to evaluate trustworthiness, through assessing credibility, transferability, conformability, and dependability of the qualitative data. The codded data were checked by participants, peers and the experts and their feedback were considered.

## The review process

Then an extensive review of literature was performed using key words include: “scale”, “tool”, “instrument”, “check list”, “questionnaire”, “gender”, “maternity”, “perinatal”, through databases, PubMed, Google Scholar, Science Direct, Scopus, and World Health Organization. Findings of this part did not add any items.

## GS-PNCS development

### Item generation

The first extracted items were generated from qualitative part of the study (72 items). The review of literature did not add any items to the preliminary questionnaire.

### Scoring

The scale was scored based on a 3-point Likert scale, scoring 1 to 3 for “not at all”, “a little” and “much” options, respectively.

## Quantitative part: psychometric assessment of GS-PNCS

In the quantitative part, the psychometric properties of the questionnaire including; quantitative and qualitative face validity, quantitative and qualitative content validity, construct validity and reliability of the tool were assessed.

### Face validity assessment

For qualitative assessment of face validity, the preliminary questionnaire was evaluated by 15 perinatal care providers and managers including; 2 reproductive health specialists, 3 perinatal service managers and 8 midwives, and 2 health experts. These participants assessed difficulty, generality and ambiguity of the items. The items impact scores were calculated to assess the face validity quantitatively. At this stage, the above mentioned participants rated each item by the 5-point Likert scale from completely important to not at all important, scoring 5 to 1. To calculate the item impact score, the following formula was applied: ***Item impact score = Frequency (percentage) × Importance***. The items with an impact score of more than 1.5 were appropriate and maintained for later stages [22].

### Content validity assessment

Content validity of the questionnaire was examined qualitatively and quantitatively. In the qualitative content validity assessment, 10 experts in reproductive health and midwives were asked to comment on the items regarding the grammar of items, choice of vocabulary, placement of items, and scoring [23]. In quantitative content validity assessment, content validity ratio (CVR) and content validity index (CVI) were calculated.

The content validity ratio was assessed by 13 experts. The participants scored the items based on a 3-point Likert scale (essential, useful but not essential, not essential).

CVR was calculated through the following formula

$$ CVR=\frac{n_E-\left(N/2\right)}{N/2} $$

where *n*_*E*_ stands for the number of specialists who have chosen the option “essential” and *N* is the total number of specialists. According to Lawshe’s table [24], the CVR higher than 0.54 for 13 individuals indicate the necessity of the item at a statistically significant level (*P* = 0.05).

Content Validity Index (CVI) was assessed by the same 13 experts who scored items of the questionnaire based on their "simplicity", "relevance" and "clarity" using the 4-level Likert scale (scores 0 to 3 for “not at all” to “completely”) based on Waltz & Bausell’s content validity index [25].

CVI was calculated according to the following formula:

$$ \mathrm{CVI}=\frac{\ \mathrm{Number}\ \mathrm{of}\ \mathrm{raters}\ \mathrm{chosing}\ \mathrm{points}\kern0.5em 3\ \mathrm{and}\kern0.5em 4}{\ \mathrm{Total}\ \mathrm{number}\ \mathrm{of}\ \mathrm{raters}} $$

Items with a CVI higher than 0.79, between 0.70 and 0.79, and lower than 0.70 were considered suitable, needing modification, and unacceptable, respectively [23]. The scale’s content validity ratio (S-CVR) and the scale’s content validity index (S-CVI) were obtained through calculating of mean of items’ CVR and CVI.

### Construct validity assessment

#### Design of the study

Construct validity of GS-PNCS was evaluated through exploratory factor analysis (EFA) through a cross sectional study.

#### Subjects of the study

285 PNCs’ providers including prenatal, child birth and postpartum care providers, with at least 2 years of care or management experience, and willingness to participate were recruited.

#### Sampling

The number of samples in this study was determined 5 samples for each item of the designed questionnaire. Plichta et al. (2013) states that the required number of responders for EFA is between 3 and 10 persons per item, or a total of 100 to 200 responders [26]. Therefore, all 285 perinatal care providers of health centers and hospitals affiliated to Shiraz University of Medical Sciences in Iran, who had the inclusion criteria of the study recruited for the study using convenience method of sampling.

#### Setting

The subjects of the study were recruited from all 37 health centers and all 9 public and private hospitals in Shiraz.

#### Tool for data collection

Tool for data collection was GS-PNCS following face and content validity assessment.

#### Data analysis

To confirm the adequacy of the sample size for EFA, two criteria of Kaiser-Meyer-Alekin (KMO) and Bartlett Sphericity Test were measured. Adequacy of the sample size for EFA could be shown while the calculated KMO index is more than 0.8 and the *p* value of the Bartlett Sphericity test is less than 0.05 [26].

Then, the items were examined regarding suitability to enter factor analysis by calculating of commonalities. Next, items with commonalities of higher than 0.4 were selected for the analysis.

Quartimax rotation was used for factor analysis in this study. *Quartimax minimizes* the *number* of *factors needed* to *explain* each *variable* [27]*.* The factors of the tool were extracted using the Kaiser (1960) criterion, with the acceptance of factors having an Eigen value of more than one; and drawing the Screeplot.

### Reliability assessment

To confirm the GS-PNCS’s reliability, internal consistency was assessed through Cronbach’s alpha calculation, and the questionnaire’s stability was evaluated through the calculation of the correlation coefficient of the test–retest and the intraclass correlation coefficient.

#### The internal consistency assessment

*The internal consistency* of GS-PNCS was calculated by calculating the Cronbach’s alpha coefficient, and the values above 0.7 were considered acceptable [28, 29]. To assess internal consistency, 30 eligible providers of perinatal care filled up the questionnaire

#### Stability assessment

Stability of GS-PNCS was assessed through the test–retest method. The questionnaires were filled up by 30 eligible providers with a 2-week interval and then Pearson correlation and intra-class correlation coefficients of scores of the two tests were calculated.

The Pearson correlation coefficient more than 0.7 [23] and ICC higher than 0.4 were considered as the acceptable levels for stability [30].

**Fig. 1:**
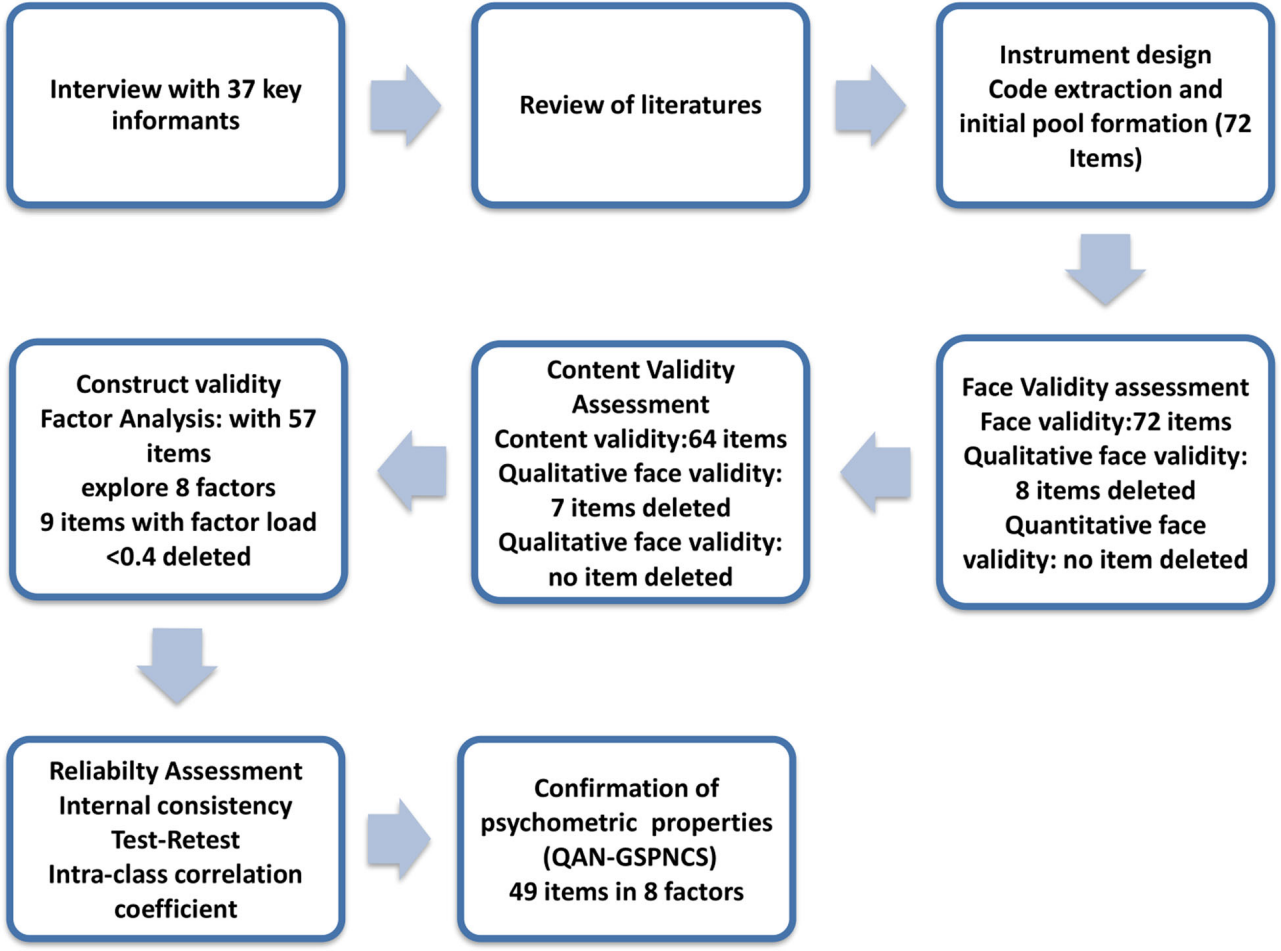
Process of designing and assessment of Psychometric properties of the GS-PNCS

The SPSS-V.21 was used to perform all statistical analyses. A summary of steps for designing and assessment of psychometric properties of GS-PNCS is presented in Fig. 1.

## Results

The Findings are presented in two parts: 1) designing of GS-PNCS; and 2) assessing the psychometric properties of the GS-PNCS.

### Qualitative phase: designing of the questionnaire

In the qualitative section, 34 interviews with 34 perinatal care providers and manages were performed in their office or PNC clinics. Nobody refused or dropped out the interviews. Then an extensive review was performed on the *related* literature. These led to the explanation of the concept of gender sensitive PNCS. Then, using the extracted concept, the practical definitions of the dimentions of gender sensitive PNCS were extracted. Accordingly, the questionnaire of gender sensitive perinatal care services (GS-PNCS) is a tool that measures the responsiveness of PNCS to the needs of men and women based on their gender roles. The needs are in all dimensions of the services including structure of the services consisting of human resources, facilities and managers; processes of the services including care and educational procedures; supporting policies, consist of intersectoral co-operation and community empowerment.

**Fig. 2:**
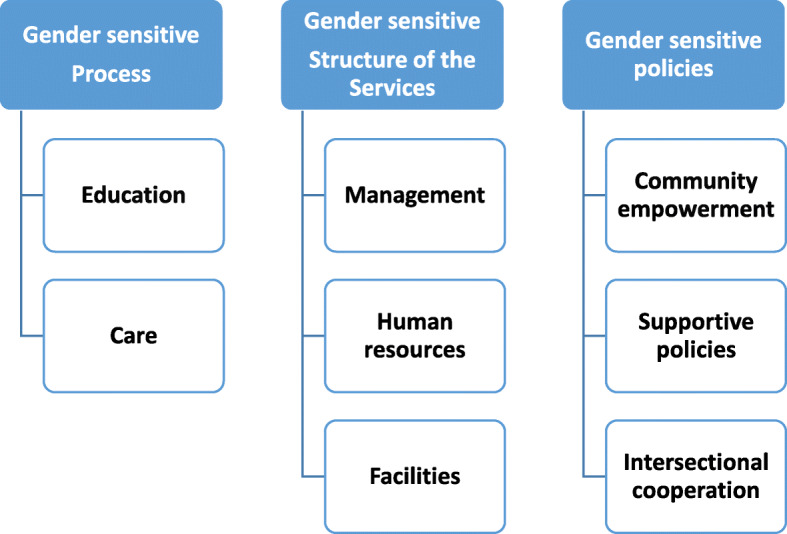
Categories and subcategories extracted from the qualitative phase for designing of GS-PNCS

The extracted items from the qualitative part and the literature review made the primary pool of GS-PNCS. These 72 items were classified in 8 subcategories and 3 categories/themes (Fig. 2).

### Quantitative phase: assessment of psychometric properties of GS-PNCS

In the quantitative part, face-, content- and construct validity and then the reliability of GS-PNCS were examined.

#### Face validity assessment

In the qualitative face validity assessment, 8 items were omitted for ambiguity and generality. In the quantitative face validity assessment, the importance of each item was measured and the items with impact score of more than 1.5 were maintained. In this stage, all phrases received a score of more than 1.5.

#### Content validity assessment

In the Qualitative Content validity assessment, 7 items were deleted. In quantitative content validity assessment, no item was deleted as they obtained acceptable CVI and CVR level. Finally, content validity of GS-PNCS was confirmed by S-CVR and S-CVI, 0.92 and 0.98, respectively.

Then, the questionnaire with 57 items entered the stage of construct validity assessment. Figure 1 shows the process of designing and assessing psychometric assessment of GS-PNCS and the related changes of the questionnaire.

#### Construct validity assessment

The EFA method was used to assess the construct validity of GS-PNCS. Sample size for this section of the study was considered 5 sample for each items. Thus, for 57 items, 285 perinatal care providers recruited for the study (Table 1). The calculated KMO index was 0.822 and the Bartlett Sphericity test showed the correlation matrix 7715.23 with *P* < 0.0001 which both showed sample adequacy for EFA.

**Table 1:** Demographic characteristics of participants for factor analysis of GS-PNCS (*n* = 285)

Characteristics	Category	Number	Percent
Age	20–30	171	60.0
	31–40	112	23.8
	> 40	42	16.2
Education	Midwife (Bachelor)	235	82.5
	Midwife (Graduate Diploma)	22	7.7
	Midwife (Master)	17	6.0
	Health educator (Bachelor)	9	3.1
Job experience (Years)	2–5	137	48.1
	6–10	78	27.3
	11–15	40	14.0
	16–25	30	10.6

Then, Commonalities calculations for items led to omission of an item “training medical students about health education of men and families” was omitted with commonality of< 0.3.

**Fig. 3:**
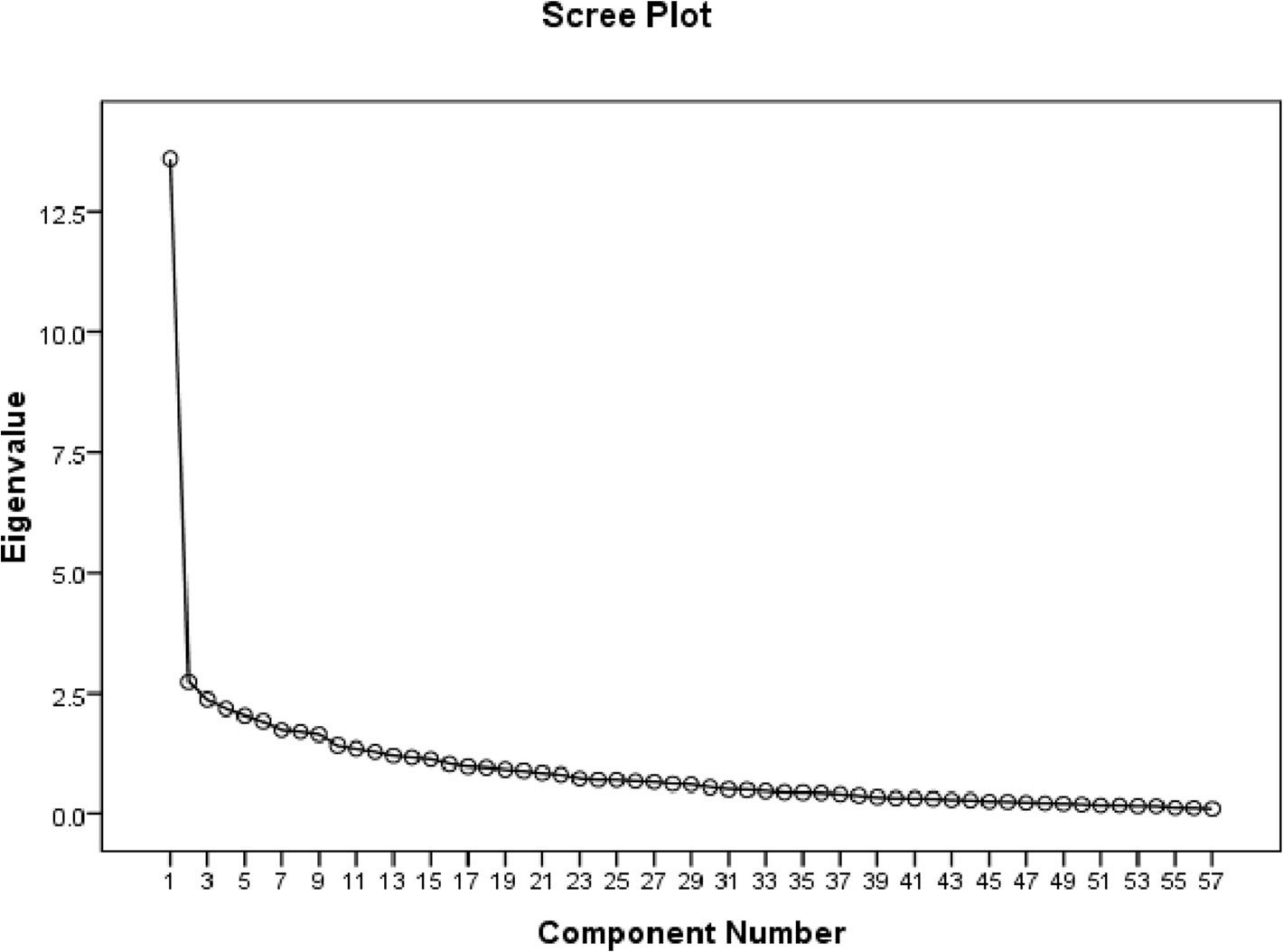
Scree plot of the exploratory factor analysis of GS-PNCS

Scree plot was used to predict the number of factors. The scree plot suggested 9 factors that became the default for factor analysis (Fig. 3)

Nine factors that explained 52.53% of cumulative variance of GS-PNCS were identified using the minimum eigenvalues of 1. After Quartimax rotation and considering the factor loading of at least 0.4, the items forming each factor were identified. Then, factor 8 was merged with factor 7 due to the fact that it contained only one item. 8 items with factor loading of 0.4 were omitted.

Table 2 reports the rotated factor matrix of the GS-PNCS.

**Table 2:** Rotated factor matrix of the GS-PNCS

Items	Factors							
	1	2	3	4	5	6	7	8
1 Planning programs for enhancing male participation in perinatal care	0.689							
2 Education about preventing son preference in schools	0.622							
3 Women’s education about male participation in perinatal care	0.617							
4 Planning to help single pregnant women	0.573							
5 Educating men about problems related to unwanted pregnancy and abortion	0.567							
5 Correcting women’s misconceptions about male participation in perinatal care	0.558							
6 Adolescents’ education about the risks of pregnancy and abortion	0.553							
7 Academic researches to eliminate son preference	0.541							
8 Gaining support from policy makers to promote the male participation in perinatal care	0.540							
9 Develop community educational programs to promote male participation in perinatal care	0.526							
10 Promotion of male participation in perinatal care services through the media	0.499							
Teaching “Parenting” in schools	0.493							
Considering male personnel to provide perinatal services to men	0.462							
Training male perinatal care providers to respond to men’s problems	0.416							
Promoting male participation for making informed decision about method of childbirth	0.405							
Promoting men’s awareness about methods of childbirth	0.402							
Devoting appropriate time for men’s perinatal services		0.693						
Engaging volunteers to help in providing “parenting” services		0.626						
Employment of personnel to provide services to without any discrimination for men or women		0.518						
Providing services for diagnosis, treatment and follow up of male sexually transmitted diseases		0.478						
Providing counseling services for high-risk sexual behavior cases		0.447						
Providing sexual health counseling services by trained personnel		0.409						
Considering appropriate physical conditions for men’s attendance in perinatal services			0.699					
Integrating prenatal health comments in premarital counseling programs			0.613					
Planning to correct misbeliefs of the providers about male participation providers about male participation			0.605					
Providing friendly care services for pregnant women with AIDS			0.554					
Recommending condom use to men with high-risk sexual behaviors			0.489					
Management of workload for integrating paternal services in perinatal care services			0.442					
Education of reproductive health rights in universities				0.668				
Developing guidelines to protect rights of pregnant mothers in temporary marriage				0.552				
Girls’ education about the risks of pregnancy and abortion in schools				0.491				
Training care providers about sexual health and rights of pregnant mothers				0.478				
Developing guidelines to protect abused pregnant women				0.440				
Providing effective counseling for post-abortion clients					0.686			
Providing premarital counseling about risks of adolescents’ pregnancy for teenage couples					0.598			
Providing counseling to men about paternal role					0.419			
Providing counseling for post-abortion clients						0.676		
Providing special care and counseling before and after HIV testing of parents						0.691		
Scheduling perinatal care visits for men						0.534		
Evaluation of men’s health in perinatal care services						0.423		
Defining the Indices for Men’s Participation for quality of care assessment						0.401		
​​ Increasing personnel’s awareness about male participation							0.551	
Paternal needs assessment using indicators							0.530	
Counseling for solving paternal adaptation problems							0.522	
Monitoring the performance of the private sectors in promoting male participation							0.514	
Couple’s training about methods of childbirth							0.409	
Educating couples about the effect of partner’s sexual high-risk behaviors on maternal and fetus health								0.516
Men’s education about sexual health by educational booklets								0.490
Providing special sexual health education for pregnant adolescents								0.593

The factors 1 to 8 named as “Supportive policies to promote the gender sensitive services”; with 16 terms (explaining 14.18% of variance); “Structural reforms” with 6 items (explaining 6.57% of variance); “management considerations” with 6 items (explaining 5.83% of variance); “Women’s rights promotion” with 5 items (explaining 4.99% of variance); “educational considerations” with 3 items (explaining 4.78% of variance); “care considerations” with 5 items (explaining 4.75% of variance); “facilitating participation” with 5 items (Factor 7 and 8 totally explaining 7.74% of variance); “Sexual Health education” with 3 items (explaining 3.94% of variance); respectively.

Table 3 demonstrates the comparison of dimensions and sub-scales in qualitative and quantitative findings in the mix study of gender sensitive perinatal care services.

**Table 3:** The comparison of dimensions and sub-scales in qualitative and quantitative findings in the mix study of gender sensitive perinatal care services

Dimensions	Categories (Qualitative study)	Sub-Scales (Factor Analysis)
Gender Sensitive Policies	Supportive policies	Supportive policies to promote gender sensitive services
	Community empowerment	
	Intersectional cooperation	Women’s rights promotion
Gender Sensitive Structure	Human resources	Structural reforms
	Facilities	Facilitating male participation
	Management	Management considerations
Gender Sensitive Process	Care	Care considerations
	Education	Educational considerations
		Sexual health Education

#### Reliability

To ensure reliability, both internal consistency and stability of GS-PNCS were assessed. Internal consistency of GS-PNCS was demonstrated by Cronbach α at 0.880 for whole instrument. To investigate stability, using the test–retest method, the correlation between the two testing occasions was computed. Correlation coefficient and intraclass correlation coefficient of the whole questionnaire were reported 0.980 and 0.973, respectively. Table 4 Displays the results of questionnaire’s reliability assessment. After confirming validity and reliability of the GS-PNCS, the questionnaire was finalized.

**Table 4:** Stability Coefficients and Interclass Correlation Coefficient of the GS-PNCS Subscales

Factors	Cronbach’s α coefficient	Interclass correlation coefficient	Test–retest Pearson correlation coefficient
Supportive policies to promote gender sensitive services	0.905	0.817	0.895
Women’s rights promotion	0.952	0.956	0.968
Structural reforms	0.780	0.927	0.927
Faclitating male participation	0.864	0.916	0.948
Management considerations	0.836	0.977	0.979
Care considerations	0.889	0.971	0.972
Educational considerations	0.896	0.973	0.980
Sexual Health education	0.889	0.991	0.999
Total	0.880	0.973	0.980

*Note.* PAQ = Paternal Adaptation Questionnaire

#### Scoring procedure by GS-PNCS

GS-PNCS was scored by a rating scale 1 to 3. The range of scores for the whole questionnaire and its subscales are presented in Table 5. The total score of the GS-PNCS and its subscales are calculated and presented as percentages. The range of scores is from 49 (0%) to 147 (100%) describing adequate gender sensitive PNC services to completely non adequate PNC services.

**Table 5:** The Range of Scores and Subscales of the GS-PNCS

Factors/Subscales^a^	NO Items	Range of scores
Supportive policies to promote the gender sensitive services	16	16–48
Women’s rights promotion	5	5–15
Structural reforms	6	6–18
Facilitating male participation	5	5–15
management considerations	6	6–18
care considerations	5	5–15
educational considerations	3	3–9
Sexual Health education	3	3–9
Total	49	49–147

*Note.*GS-PNCS = Questionaaire to assess Gender Sensituve Pernatal Care Services*;*^a^The score of total and the subscales are calculated and presented as percentage

#### Description of GS-PNC

GS-PNCS is a valid scale with 49 items and 8 subscales that can be scored fron 49 to 147 (o to 100%) and measures needs for a gender sensitive perinatal care service and its higher scores shows higher needs for the gender based PNCS.

Table 6 shows final version of the GS-PNCS with 49 items after Psychometric Properties Assessment.

**Table 6:** The GS-PNCS at the End of Psychometric Properties Assessment

Please show your opinion (by √) about the following needs for your perinatal care services to be gender sensitive (GS-PNCS)?				
How much the following "supportive policies" are necessary?		Not at all	Little	Much
1	Gaining support from policy makers to promote the male participation in perinatal care			
2	Develop community educational programs to promote male participation in perinatal care			
3	Promotion of male participation in perinatal care services through the media			
4	Correcting women’s misconceptions about male participation in perinatal care			
5	Women’s education about male participation in perinatal care			
6	Educating men about problems related to unwanted pregnancy and abortion			
7	Adolescents’ education about the risks of pregnancy and abortion			
8	Teaching "Parenting" in schools			
9	Education about preventing son preference in schools			
10	Academic researches to eliminate son preference			
11	Planning to help single pregnant women			
12	Planning programs for enhancing male participation in perinatal care			
13	Considering male personnel to provide perinatal services to men			
14	Training male perinatal care providers to respond to men’s problems			
15	Promoting men’s awareness about methods of childbirth			
16	Promoting male participation for making informed decision about method of childbirth			
**How much the following strategies for "Women’s rights promotion" are necessary?**				
17	Developing guidelines to protect abused pregnant women			
18	Developing guidelines to protect rights of pregnant mothers in temporary marriage			
19	Training care providers about sexual health and rights of pregnant mothers			
20	Girls’ education about the risksof pregnancy and abortion in schools			
21	Education of reproductive health rights in universities			
**How much the following "Structural reforms" are necessary for the perinatal care services?**				
22	Devoting appropriate time for men’s perinatal services			
23	Providing services for diagnosis, treatment and follow up of male sexually transmitted diseases			
24	Providing counseling services for high-risk sexual behavior cases			
25	Providing sexual health counseling services by trained personnel			
26	Employment of personnel to provide services to without any discrimination for men or women			
27	Engaging volunteers to help in providing "parenting" services.			
**How much the following strategies are necessary to "Facilitating male participation"?**				
28	​​ Increasing personnel’s awareness about male participation			
29	Paternal needs assessment using indicators			
30	Counseling for solving paternal adaptation problems			
31	Monitoring the performance of the private sectors in promoting male participation			
32	Couple’s training about methods of childbirth			
**How much the following "management considerations" are necessary for gender sensitive Perinatal services?**				
33	Considering appropriate physical conditions for men’s attendance in perinatal services			
34	Integrating prenatal health comments in premarital counseling programs			
35	Planning to correct misbeliefs of the providers about male participation			
36	Providing friendly care services for pregnant women with AIDS			
37	Recommending condom use to men with high-risk sexual behaviors			
38	Management of workload for integrating paternal services in perinatal care services			
**How much the following "care considerations" are necessary for gender sensitive perinatal care services?**				
39	Scheduling perinatal care visits for men			
40	Evaluation of men’s health in perinatal care services			
41	Defining the Indices for Men’s Participation for quality of care assessment			
42	Providing care for post abortion patients			
43	Providing special care and counseling before and after HIV testing of parents			
**How much the following "educational considerations" are necessary for gender sensitive perinatal care services?**				
44	Providing counseling to men about paternal role			
45	Providing premarital counseling about risks of adolescents’ pregnancy for teenage couples			
46	Providing counseling for post-abortion clients			
**How much the following "sexual health education" are necessary in the perinatal care services"**				
47	Educating couples about the effect of partner’s sexual high-risk behaviors on maternal and fetus health			
48	Men’s education about sexual health by educational booklets			
49	Providing special sexual health education for pregnant adolescents			

## Discussion

GS-PNCS is **the first tool to assess gender sensitivity** and appropriateness of PNCS for men and women. This valid and reliable tool is able to measure the responsiveness of perinatal services to the gender specific needs and so helps health care managers and planners to improve the quality of PNCS. Gender equity is mentioned as the characteristics of quality of maternal care services [31] and GS-PNCS is able to evaluate the adequacy of perinatal care responsiveness to clients’ needs based on their gender roles.

GS-PNCS is a **valid and important assessment tool** to measure quality of PNCS regarding their gender sensitivity and so useful to improve the quality. A few tools are developed and applied to assess quality of PNCS [32–35], however, they are not able to measure the gender sensitivity of the services and moreover these tools were not assessed regarding some aspects of psychometric properties especially construct validity.

GS-PNCS was designed by inductive-deductive approach [19]. The qualitative part and the literature review demonstrated **8 dimentions** for gender sensitive perinatal services including; gender sensitive care and educarional process; gendersensitive facilities, human resources and mangement; and community empowerment, sopurtive policies and intersectoral cooperation for the gender sensitive services. Then, **the EFA indicated eigth factors** which six of them were similar to the dimentions of the qualitative part. Finding showed PNCS need managerial, structural, facilities, educational and care procedures reforms for respoding to the specific needs of genders especially men which should be supported by the supportive policies [36, 37].

Face and content validity of GS-PNCS was confirmed qualitatively and quantitatively. Proper validity of a questionnaire usually refers to the vision of the target group about face validity, suitability, attractiveness, comprehensibility, culturally and socially appropriateness, logically sequence of the elements and the completeness of the instrument [19]. In qualitative face validity 8 items were deleted due to vague and duplication. In the quantitative face validity assessment, impact score of all items were higher than 1.5 and shown to be acceptable. Content validity of GS-PNCS was also confirmed by S-CVR and S-CVI 0.92 and 0.98, respectively. It shows that GS-PNCS has an appropriate sample of items for measuring gender sensitivity of the services [38].

Results of EFA showed “Supportive policies to promote the gender sensitive services”; with 16 terms explains highest variance and predictability for the sensitivity of the services “Structural reforms” and “management considerations” were second and third factors regarding their predictability for sensitivity of the services. Reproductive health policies and program formulation, has generally relied on data collected from women, while adequate policies and strategies are necessary to both men and women in their fertility control and STIs prevention care services [39]. Therefore, supportive policies are necessary as studies demonstrated that policy makers can increase implementation and effectiveness of an innovation such as making the services gender sensitive, by concentration on creating an environment that the providers perceive importance of the providing gender based services. In addition, managers should consider specific structural changes to increase positive perceptions and condition for implementation [40]. Gender roles are influenced by cultural characteristic of different communities. Besides, optimal patient care is affected by both scientific and social characteristics [41]. Therefore, community supportive policies and then structural changes by the gender sensitive management are necessary to improve Health services [42].

“Women’s rights promotion” and “Facilitating male participation” were also extracted as the subscales of GS-PNCS. Studies shows that Reaching men to end gender-based violence and promote sexual and reproductive health rights of women are necessary [43]. Because of unequal gender–power relations, women are especially vulnerable but are often unable to negotiate changes in sexual behavior or to practice safe sex without the cooperation of their sexual partners. Therefore, men participation in reproductive health can span several themes. For example, men can be sources of transmission of STIs to women. When women get pregnant, their partners participate in making decisions which affects on their pregnancy such as seeking health care and place of delivery [44]. Therefore, special efforts should be made to emphasize men’s shared responsibility and promote their active involvement in responsible sexual and reproductive behavior. It seems reasonable that if men are brought into a wide range of reproductive health services in such a way that they are supported as equal partners and responsible parents, as well as clients in their own right, better outcomes will be observed among both women and men [45].

Educational and care considerations based on gender specific needs and providing the sexual health education were other predictors of gender sensitivity of the services. The items were mainly related to providing care and education and sexual health services for men. Male involvement in perinatal care led to better birth outcomes. However, men are usually unavailable to attend perinatal programs because of work or feeling unwelcome at programs deemed “only for women” [46]. While they need care and education regarding their fatherhood adaptation process and roles during perinatal period [36]. Appropriate preparation for fatherhood has the potential to enhance maternal, child, and family health and even educational media such as e-health provide opportunities for men to prepare for fatherhood [37, 47, 48].

Internal consistency and stabilityof GS-PNCS suggest high reliability of the questionnaire for assessment of gendersisivity of the questionnaire.

The study defined the concept of gender sensitivity of PNCS as a variable that can be measured by GS-PNCS containing 8 subscales that predict 53% of variance. Finding demonstrated gender sensitive PNCS needs supportive policies at the first steps and then requires the structural reforms by some management actions and considering some reforms in care and educational procedures and providing sexual health services. Meanwhile promotion of women right and male participation both in community and in the services are necessary. GS-PNCS provides the criteria for making PNCS gender sensitive and can be a base for the reform of the services.

GS-PNCS is developed and validated to show gaps in PNCS in the health care system of Shiraz. However, regarding to high validity and reliability of the questionnaire, it can be utilized not only in Shiraz but also for similar health care system of other provinces in Iran. Also it can be used to evaluate quality of PNCS in health systems of other countries. However, its validity and reliability is recommended to be assessed after translation to other languages.

GS-PNCS is a valid and reliable tool to show gaps in structure and procedures of PNCS and so helps to show priorities for the necessary interventions for planning a comprehensive gender based quality PNCS.

## Conclusion

GS-PNCS is a valid (S-CVR = 0.92 and S-CVI = 0.98) and reliable (Cronbach’s α = 0.880 and the test-retest and Pearson Correlation = 0.947 and ICC = 0.980) questionnaire with 49 items to assess gender sensitivity of PNCS by a three level Likert scale. It include with 8 subscales including; “Supportive policies to promote the gender sensitive services”; “Structural reforms” “management considerations”; “Women’s rights promotion”; “educational considerations”; “care considerations”; “Facilitating male participation”; “Sexual Health education” which predict 52.53% of variance.

## Data Availability

The datasets generated and/or analysed during the current study are not publicly available because the data are a part of an extensive research on reproductive gender sensitive reproductive health services including PNC, but are available from the corresponding author on reasonable request.

## Abbreviations

STIs: Sexually transmitted infections
PNCS: Perinatal care services
GS-PNCS: Gender sensitivity of perinatal care services
S-CVR: Scale-level content validity ratio
S-CVI: Scale-level content validity index
CVR: Content Validity Ratio
CVI: Content Validity Index
ICC: Intra-class correlation
EFA: Exploratory factor analysis
KMO: Kaiser-Meyer-Alekin
